# Recurrent neck abscesses due to cervical tuberculous lymphadenopathy in an elderly woman post-splenectomy: a case report

**DOI:** 10.1186/1752-1947-5-584

**Published:** 2011-12-20

**Authors:** Aaron L Niblock

**Affiliations:** 1 Royal Victoria Hospital, Medical Department, 274 Grosvenor Road, Belfast BT12 6BA

## Abstract

**Introduction:**

There are approximately 7000 new cases of tuberculosis every year in the UK, the majority of which are pulmonary. Approximately 5% affect the lymph nodes in immunocompetent patients. Scrofula is an old term used to describe lymph nodes of the neck infected with tuberculosis

**Case presentation:**

In the elderly population, growing neck lumps are always treated as red flags until a diagnosis is confirmed. Here, the case of an 89-year-old Caucasian woman is presented. She was reluctant to seek medical help as she feared the cause was sinister and did not want surgical intervention.

**Conclusion:**

It is difficult to culture tuberculosis from superficial swabs, resulting in a high proportion of false negative results. Where there is a high degree of clinical suspicion for tuberculosis, it is important to consider a biopsy with culture. Patients over the age of 65 have waning immunity and are therefore a vulnerable group for acute infections as well as the re-activation of indolent organisms. Post-splenectomy patients are at a major disadvantage during sepsis and when a cellular immune response is required, such as when faced with a *Mycobacterium tuberculosis *infection. Scrofula is treated with a similar regime as pulmonary tuberculosis and has a near 100% success rate.

## Introduction

In the elderly population, growing neck lumps are always treated as red flags until a diagnosis is confirmed. The case of an elderly patient presenting with neck lumps is here described. She was reluctant to seek medical help as she feared the cause was sinister and did not want surgical intervention. There are many laboratory tests used to diagnose tuberculosis (TB) infection however we rely on a high level of clinical suspicion.

Scrofula is an old term for TB affecting the lymph nodes of the neck, a more meaningful term is cervical tuberculous lymphadenopathy. It is usually the result of a primary infection of the lymph nodes with *Mycobacterium tuberculosis*. The bacteria can be spread by the lymphatic system or blood. Therefore, it could originate from a primary pulmonary focus. In adults, it is usually *M. tuberculosis *and in children, nontuberculous mycobacteria [1].

Scrofuloderma results from the breakdown of skin overlying a tuberculous focus, usually at a lymph node but also where skin overlies infected bones or joints. In the past, milk was frequently contaminated with *M. bovis *that resulted in a high number of children presenting with scrofuloderma. The oral or tonsillar primary lesion could progress to cervical adenitis which in turn resulted in the formation of cold abscesses. Clinically, these lesions are firm painless nodules that gradually enlarge and suppurate, forming ulcers and sinus tracts in overlying skin. Spontaneous healing can occur but often takes years and is often accompanied by the formation of hypertrophic scars. As in this case, the most common presentation of scrofula is a painless, often suppurative abscess that shows no signs of calor or erythema unless there is a secondary infection. Patients less frequently present with systemic signs such as weight loss and night sweats [2].

In Europe, with the rapid decrease of TB in the second half of the twentieth century, scrofula has become a very rare disease. The marked decrease in prevalence amongst the more economically developed countries was due to the pasteurization of milk and the Bacillus Calmette-Guérin vaccine. TB is still a major problem across the less economically developed countries, and therefore clinicians have a higher degree of suspicion when assessing patients.

**Figure 1:**
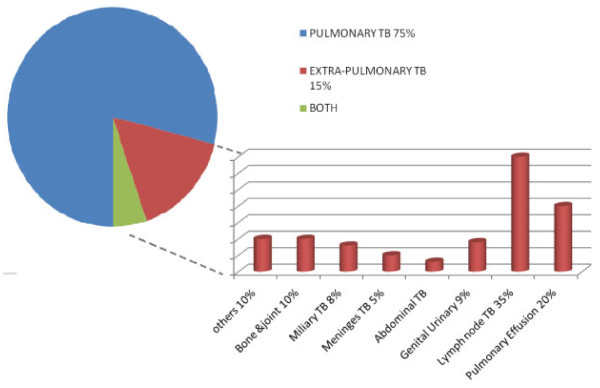
**Pie Chart to compare the % of Pulmonary TB (PTB) to Extra-Pulmonary TB (EPTB) in newly diagnosed cases**. Column graph demonstrates the breakdown of Extra Pulmonary sites of TB infection in the 15-20%.

Every year in the UK, it is estimated that there are 7000 new cases of TB. According to a study carried out in India, extrapulmonary TB constitutes about 15% to 20% of new TB cases in immunocompetent patients. These extrapulmonary sites are demonstrated in Figure 1. This can rise to over 50% in immunocompromised patients, especially in patients positive for the human immunodeficiency virus (HIV) [3].

### Body response to infection with TB

TB is spread by droplets that are inhaled into the respiratory tract, passing through the pharynx. It most commonly affects the lungs although it is possible to trigger a response in the tonsillar region. How the host's immune system responds to *M. tuberculosis *has a major role in determining the clinical manifestations. Once the organism has reached the alveoli it has four potential fates [4]: the immune system may destroy the bacilli and the patient gains immunity; the bacilli multiple and cause disease, that is, pulmonary TB; the bacilli become dormant and never cause disease; or the patient may develop reactivation TB - active disease due to an existing impairment in the immune system through, for example, HIV, malignancy or malnutrition.

During the primary infection with TB and during any subsequent secondary active disease, the bacteria are spread by blood or through the lymphatic system to any part of the body. Usually the bacteria are destroyed by the immune system; however, they may concentrate at a particular site and lie dormant for decades before causing disease.

## Case presentation

**Figure 2:**
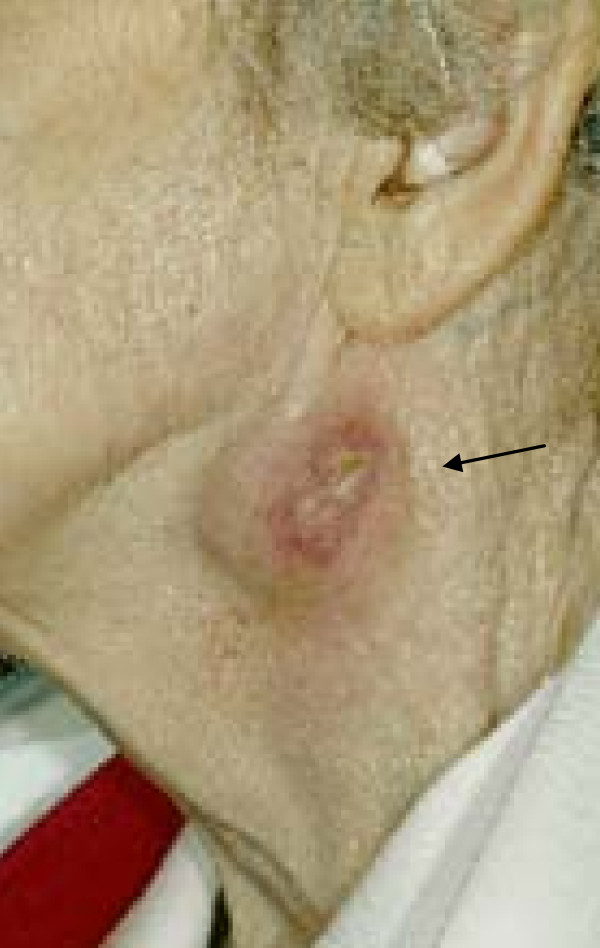
**Image to show left sided neck abscess**.

An 89-year-old Caucasian woman presented to our outpatients clinic with a left-sided neck lump which she had had for six months. It had started as a small pea-sized lump and developed into a 3 cm × 4 cm smooth, fixed mass (Figure 2). It had grown relatively slowly and the woman has been otherwise healthy throughout. She reported no pyrexia, dysphagia, weight loss, night sweats or hoarseness. Accompanied by her niece, our patient made it clear that, if it was malignant, she wanted it treated conservatively.

A further history revealed that she had a three-year history of recurrent neck swellings that progressed to abscesses, continuously discharging and healing slowly.

**Figure 3:**
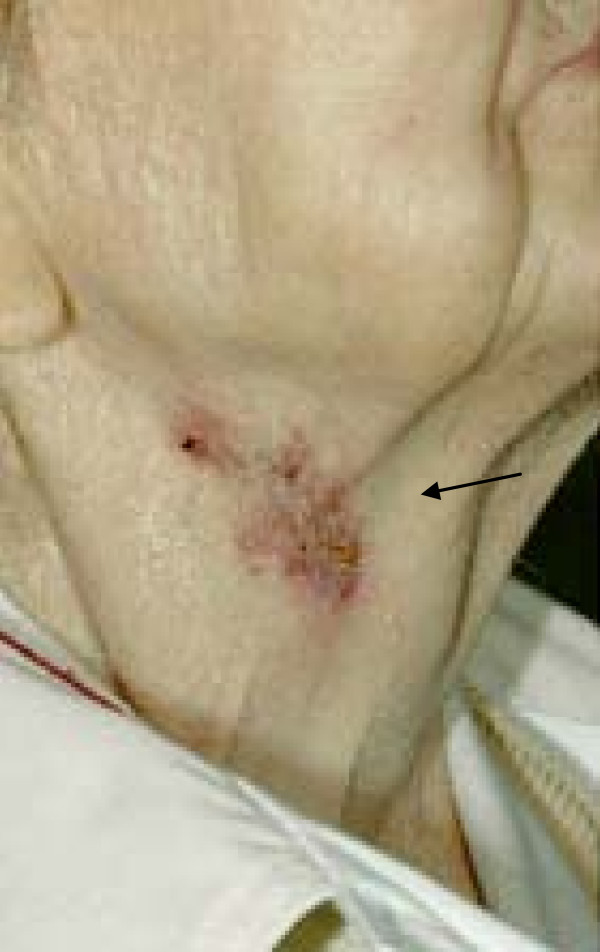
**old slow to heal right sided neck abscess, scrofuloderma skin changes**.

**Figure 4:**
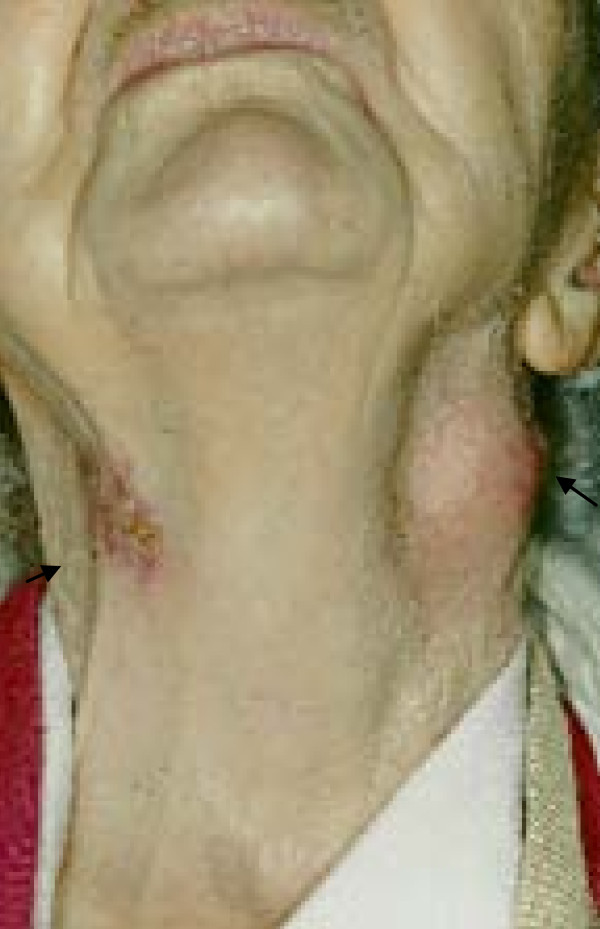
**image to show the contrast between the two abscess at different stages, left sided is more recent whilst the right has partially healed with the surrounding scrofuloderma skin changes**.

On closer physical inspection, there was visible scarring from a previous abscess approximately 5 cm inferior to this mass on her left side. As can be seen in Figure 3 and Figure 4, on our patient's right side there was an old, slowly healing abscess that has been present for over a year.

Our patient had always enjoyed good health, with the only other significant past medical history being a splenectomy, resulting in taking life-long daily penicillin. The abscesses were treated by her general practitioner with recurrent antibiotics and frequent dressings. Previous swabs from the neck abscess, sent by her general practitioner, did not grow the tuberculous organism.

Our patient agreed to have further investigations. A chest X-ray and fine needle aspiration (FNA) were performed. The chest X-ray showed chronic fibrotic changes and no evidence of TB infection. The FNA contained necrotic debris and foam cells; acute inflammatory cells were not prominent and granulomata were not seen. Ziehl-Neelsen staining was performed and no acid-alcohol fast bacilli were seen. The features found represented a partially treated skin abscess but the negative staining did not exclude the possibility of TB. Part of the aspirate was submitted to microbiology for conventional and TB culture.

The differential diagnosis included neoplasia, tertiary syphilis, deep fungi (for example, sporotrichosis, actinomycosis) and chronic granulomatous disease.

Three weeks after submitting the aspirate to microbiology for culture, TB was confirmed. The infectious disease team took over our patient's care immediately. Our patient underwent various tests, including liver function tests, urea and electrolytes test, HIV screening and visual acuity prior to commencing the anti-TB regime.

Our patient was started on ethambutol 700 mg to be taken in the morning, rifampicin 450 mg, isoniazid 300 mg, pyrazinamide 900 mg and pyridoxine 20 mg. Within two months she showed a significant improvement, and after four months there were visible scars only.

## Discussion

The body's initial immune response involves Type 1 CD4+ T lymphocytes and natural killer T lymphocytes (NK cells) that secrete interferon-gamma. This activates macrophages to produce a variety of substances that inhibit growth and kill mycobacteria. This is a very simplified explanation; the process involves many other aspects including suppression from interleukin 4-10, amplification by interleukin 12 and many cytokines [5].

Re-infection is a rare event when immunity is intact, however, over the past few decades it has been demonstrated in patients with advanced HIV, with the use of chemotherapy and disease-modifying antirheumatic drugs and, in theory, is possible in any other process that reduces the host immune response [6].

Aging is known to have detrimental effects on the immune system and is referred to as immunosenescence. It is a complex process that affects cell mediated immunity [7]. As we age, we lose lymphoid tissue. T-cell activation is reduced plus a larger percentage of activated T-cells responses start later and stop sooner. NK cell activity is also reduced significantly. It has been suggested that, as we age, the innate response prevails over the adaptive response [8-10].

The immune response to TB greatly relies on T lymphocytes and NK cells and so the aging immune system is much less capable of responding to *M. tuberculosis*.

The spleen is an important organ in the defense against invading pathogens. It acts as a filtering system, permitting phagocytosis of bacteria by cells in the reticuloendothelial system. The spleen is also an important site capable of producing large quantities of antibodies, which has proven vital in preventing and tackling sepsis. Therefore, the major risk post-splenectomy is that of overwhelming sepsis [11].

The humoral role of the spleen has been well documented and recent studies have shown the spleen to have a significant role in cell mediated immunity. The spleen is an important organ for the differentiation and maturation of stem cells into immunocompetent B-cells. B-cells were once mostly associated with the humoral immune response. However, recent studies have shown a variety of interactions of B-cells with the cellular immune response, which is necessary against infection by *M. tuberculosis *[12].

In the future, if a mass is suspected for TB, tissue should be attained to help with the diagnosis. As in this case, the initial Ziehl-Neelsen stain came back negative, however the culture was positive. This is a common problem and emphasizes the importance of a high clinical suspicion. Having to repeat FNA is also common, especially if the mass is long standing. Some empirical antibiotics, such as macrolides, have been shown to partly treat mycobacterium organisms, therefore reducing the likelihood of getting positive cultures.

Treatment is similar to that for pulmonary disease, which is with isoniazid, rifampicin, pyrazinamide and ethambutol for two months followed by a longer course of rifampicin and isoniazid. The length of time for treatment has long been debated, with no firm consensus. The duration depends on the patient, the response to treatment, the risk of relapse and the site and tissue involved. Where there is limited lymph node involvement, treatment is usually continued for at least four months. Surgery is usually not implicated. With adequate treatment, clinical remission is practically 100%. It is recommended that people in close contact, such as family members, should undergo testing for TB.

There are numerous investigations to be taken prior to initiating anti-TB treatment. These include a full blood count, a urea and electrolytes test, uric acid analysis, liver function tests, HIV screening, chest X-ray and three sputum acid-fast bacillus smears (to ensure no pulmonary involvement). Visual acuity and fields should be documented prior to treatment.

For the first two months, a regime of four drugs is used: isoniazid 5 mg/kg, rifampicin 10 mg/kg, pyrazinamide 20 mg/kg, ethambutol 25 mg/kg and pyridoxine 10 mg once daily.

Thereafter, a three-weekly regime can be used for a following four or more months: isoniazid 10 mg/kg, rifampicin 10 mg/kg and pyridoxine 10 mg once daily [13]. Local infectious disease departments should be contacted for advice and risk stratification.

## Conclusion

Our patient and her family were convinced there was an underlying malignant process and therefore she lived with these recurrent lumps and abscesses for years, undergoing daily dressings and multiple courses of antibiotics. She was very relieved to get a diagnosis of neck lymph node TB and overwhelmed to learn that the treatment basically has a 100% success rate. There is a high false negative culture result from superficial swabs, therefore a biopsy with culture should be considered if there is a high degree of clinical suspicion.

It is important to realize that patients aged over 65 years have waning immunity and therefore are a vulnerable group for infections as well as re-activation. Post-splenectomy patients are at a major disadvantage in sepsis and also in cellular immunity, as in the required response for *M. tuberculosis *infection.

## Consent

Written informed consent was obtained from the patient as well as her next of kin for the publication of this manuscript and any accompanying images. A copy of the written consent is available for review by the Editor-in-Chief of this journal.

## Competing interests

The author declares that they have no competing interests.
